# Integrative decision-making in gynecology and obstetrics

**DOI:** 10.61622/rbgo/2026rbgo44

**Published:** 2026-05-29

**Authors:** Ana Katherine Gonçalves

**Affiliations:** 1 Universidade Federal do Rio Grande do Norte Natal RN Brazil Universidade Federal do Rio Grande do Norte, Natal, RN, Brazil.

**Keywords:** Evidence-based medicine, Clinical decision-making, Gynecology, Obstetrics

Evidence-based medicine has traditionally relied on hierarchical models that prioritize randomized clinical trials and systematic reviews as the highest standards of scientific validity. While this framework remains essential for assessing methodological rigor, its isolated application may not adequately reflect the complexity of clinical practice in gynecology and obstetrics. Many clinical scenarios, particularly those involving ethical constraints, heterogeneous populations, and long-term outcomes depend on complementary sources of evidence, including observational studies, real-world data, and patient-reported outcomes. In addition, healthcare system-related factors, such as resource availability and population-specific needs, play a fundamental role in defining appropriate clinical decisions, particularly in contexts such as the Brazilian Unified Health System (SUS – Sistema Único de Saúde).^(1-4)^

We read with great interest the ongoing discussions regarding the application of evidence-based medicine in gynecology and obstetrics. Although the hierarchy of evidence remains a valuable tool for guiding clinical recommendations, its rigid application may fail to capture the complexity of real-world practice. While it supports the assessment of internal validity, it does not fully address external validity or contextual applicability, which are essential for decision-making in diverse healthcare settings.^(1-3,5)^

In obstetrics, ethical constraints frequently limit the feasibility of randomized trials, particularly in scenarios involving maternal–fetal risk. As a result, clinical decisions often rely on well-conducted observational studies. Similarly, in gynecology, especially in reproductive medicine, menopause, and gynecologic oncology there is increasing incorporation of real-world data, patient-reported outcomes, and personalized medicine approaches. These sources of evidence provide clinically relevant insights that are not always captured in traditional randomized designs.^(1-5)^

The expanding use of large databases and artificial intelligence has further transformed evidence generation, enabling analyses in broader and more heterogeneous populations. However, these approaches introduce important methodological challenges, including issues related to data quality, residual confounding, and algorithmic bias. These limitations reinforce the need for rigorous critical appraisal before integrating such evidence into clinical practice.^(1-3,5)^

Within the Unified Health System (SUS), clinical decision-making requires balancing scientific evidence with feasibility, cost-effectiveness, and equity. In this context, the highest level of evidence does not always translate into the most appropriate decision for an individual patient. Resource constraints, access to technologies, and population characteristics must be considered when applying guideline recommendations.^(4)^

These considerations support moving beyond a rigid hierarchy toward a more integrative model of evidence, in which different sources are viewed as complementary and interpreted within their clinical and contextual framework. This approach reinforces the central role of clinical judgment and shared decision-making, promoting more effective, equitable, and patient-centered care while maintaining scientific rigor.

## Data Availability

The research data are described in the article presented.
